# Characterisation of new HIV diagnoses achieved in emergency departments using an opt-in strategy, Catalonia, Spain, July 2021 to March 2024

**DOI:** 10.2807/1560-7917.ES.2025.30.47.2400781

**Published:** 2025-11-27

**Authors:** Jordi Llaneras, Patricia Álvarez-López, Rocío de Paz, Míriam Carbó, Mireia Saura, Alexis Rebollo, Cristina Olaizola, Neus Robert, Alberto Carrillo, Cristina Ramió, Emma Picart, Josep Maria Guardiola, Àlex Smithson, Ferran Rodríguez-Masià, Connie Leey, Laura González-García, Ignacio Ferro, Mariola Michelini, Margarita Sotomayor, Ariadna Rando-Segura, Adrià Curran, Emili Gené, Òscar Miró, on behalf of the Urgències VIHgila group of the Catalan Society of Emergency Medicine (SoCMUE)

**Affiliations:** 1 Emergency Department, Hospital Universitari Vall d’Hebron, Barcelona, Spain; 2 Infectious Diseases Department, Hospital Universitari Vall d’Hebron, Barcelona, Spain; 3 Emergency Department, Hospital del Mar, Barcelona, Spain; 4 Emergency Department, Hospital Clínic, IDIBAPS, University of Barcelona, Barcelona, Spain; 5 Emergency Department, Hospital Arnau de Vilanova, Lleida, Spain; 6 Emergency Department, Hospital de Bellvitge, Hospitalet de Llobregat, Spain; 7 Emergency Department, Hospital de Mataró, Mataró, Spain; 8 Emergency Department, Hospital Trias i Pujol, Badalona, Spain; 9 Emergency Department, Hospital de Sant Pau i Santa Tecla, Tarragona, Spain; 10 Emergency Department, Hospital Dr. Josep Trueta, Girona, Spain; 11 Emergency Department, Hospital de Santa Caterina, Salt, Spain; 12 Emergency Department, Hospital de Santa Creu i Sant Pau, Barcelona, Spain; 13 Emergency Department, Hospital Esperit Sant, Santa Coloma de Gramanet, Spain; 14 Emergency Department, Hospital Verge de la Cinta, Tortosa, Spain; 15 Emergency Department, Althaia Xarxa Assistencial Universitària de Manresa, Manresa, Spain; 16 Tissue Repair and Regeneration Laboratory, Institut de Recerca i Innovació en Ciències de la Vida i de la Salut a la Catalunya Central (IRIS-CC), Vic, Spain; 17 Emergency Department, Hospital Taulí, Sabadell, Spain; 18 Emergency Department, Hospital de Viladecans, Viladecans, Spain; 19 Emergency Department, Hospital Moisès Broggi, Sant Joan Despí, Spain; 20 Microbiology Department, Hospital Universitari Vall d’Hebron, Barcelona, Spain; 21 The members of the group are listed in the Acknowledgements

**Keywords:** HIV, emergency department, advanced HIV, acute infection, diagnosis

## Abstract

**BACKGROUND:**

HIV screening strategies in alternative settings, such as emergency departments (EDs), aim to increase diagnosis of occult infections and achieve 95–95–95 targets for 2030.

**AIM:**

To assess the effectiveness of an opt-in HIV screening strategy in EDs based on six clinical scenarios from a 2020 Spanish consensus document, while examining patient characteristics and linkage to-care.

**METHODS:**

This descriptive, multicentre, retrospective study analysed epidemiological, clinical, and linkage-to-care data of individuals aged ≥ 18 years newly diagnosed with HIV between July 2021 and March 2024 in 17 EDs covering 73% of the population in Catalonia, Spain.

**RESULTS:**

From 23,105 HIV serologies performed, there were 172 new diagnoses (positivity rate: 0.7%). Of these, 88.4% (152/172) were assigned male at birth, had a median age of 39 years (IQR: 30–50), and 47.9% (81/169) were Spanish. Sexual transmission was reported in 75.6% (130/172) of cases, with 55.5% (81/146) heterosexual. Fiebig stage data, available in 78.5% (135/172) of cases, showed 57.8% (78/135) had acute infection. Advanced HIV was found in 24.2% (30/124). Diagnoses related to the six clinical scenarios accounted for 54.6% (94/172) of cases. For all new diagnoses, 82.0% (137/167) were linked to specialised healthcare and started antiretroviral treatment within 9 days (IQR: 4–17), with no significant differences regarding urban/rural hospital coverage areas.

**CONCLUSION:**

An opt-in HIV screening strategy in the ED is feasible and effective, especially in detecting highly transmissible patients with acute infection. However, one in five newly diagnosed individuals remained untreated, highlighting the need for improved linkage to care.

**Key public health message**

**What did you want to address in this study and why?**

Current HIV testing strategies are not enough to uncover hidden infections. This study aimed to address the low HIV diagnosis rate in emergency departments, since many infections remain undetected in people who do not access specialised testing services. Understanding how targeted testing could improve early detection is important for reducing transmission and improving patient outcome.

**What have we learnt from this study?**

This study shows that implementing HIV testing in non-specialised settings, such as emergency departments, guided by specific clinical recommendations, can help to meet the World Health Organization's 2030 targets in terms of detection rates, linkage to care and early treatment initiation. This is particularly important for people who are less likely to undergo regular screening, such as heterosexual men.

**What are the implications of your findings for public health?**

Together with previous research, the results of this study suggest that testing people, who have specific symptoms, for HIV in emergency departments can help to find many cases that would otherwise go underdiagnosed. This would enable people to start treatment earlier, improving their health and reducing the transmission of HIV.

## Introduction

To date, human immunodeficiency virus (HIV) infection remains a global health problem, with an estimated 39.9 million (36.1–44.6 million) people with HIV (PWH) at the end of 2023 [1], of whom 1.3 million (1–1.7 million) were newly infected in 2023. Worldwide, up to 14% of PWH are unaware that they have the infection or are not receiving antiretroviral treatment (ART). This thereby leads to considerable rates of HIV-related morbidity and mortality among PWH, mostly due to late-stage diagnosis [1]. The diagnosis of HIV in the AIDS stage continues to be a relevant issue, especially in some population groups, such as women, heterosexuals and people older than 50 years [2,3]. Furthermore, undiagnosed HIV is a major contributor to the perpetuation of the epidemic, despite only 14% of PWH being the source of around 54% of new infections every year [4-7]. The findings of the present study refer specifically to the Spanish and Catalan context where the epidemiological surveillance of HIV is conducted by the Spanish Ministry of Health through the National Epidemiology Centre and its related divisions at national level, and by the Centre d’Estudis Epidemiològics sobre les Infeccions de Transmissió Sexual i Sida de Catalunya (CEEISCAT) in Catalonia, which publish annual reports [8,9].

To eliminate HIV infection, the Joint United Nations Programme on HIV/AIDS (UNAIDS) established the 95–95–95 targets for 2030 [10] aimed at ensuring that 95% of the HIV-positive population is diagnosed, 95% of those diagnosed are receiving ART and 95% of those receiving ART have a suppressed viral load (VL) [10]. The implementation of programmes to accomplish these targets has enabled high-income countries to come closer to achieving these objectives in recent years, with estimated figures of 92.5–96.6–90.4 being reported in Spain in 2021 [11]. Nonetheless, despite only 7.5% of PWH in Spain remaining undiagnosed, there is still room for improvement; screening strategies to increase the diagnosis of occult infections have been proposed in innovative settings such as emergency departments (EDs) [12-16]. Emergency departments have already been postulated as cost-effective units for opportunistic screening of haematogenous viral infections such as hepatitis B and C through several programmes [17,18]. In many countries, EDs are universally accessible and are often the only point of contact with the health system by the global population and, specifically, for the most vulnerable collectives (transgender people, sex workers, migrants, homeless or people using drugs or with mental illnesses) who are often not linked to healthcare [19].

In line with this opportunity for improving HIV diagnosis rates, the Spanish societies of emergency medicine (SEMES) and AIDS (GESIDA) published a consensus document in 2020 recommending an opt-in strategy for HIV screening in EDs for six clinical scenarios. Each of these clinical scenarios are either considered to be of high prevalence in PWH, or their practice is associated with a high risk of HIV acquisition. They include (i) community-acquired pneumonia (CAP) in the population aged 18–65 years, (ii) herpes zoster in the population aged 18–65 years, (iii) suspected sexually transmitted infection (STI), (iv) the practice of chemsex, (v) HIV post-exposure prophylaxis initiation, and (vi) mononucleosis syndrome [20]. With the support of Gilead Sciences, the Spanish national programme ‘Deja tu huella’ (Leave your mark) and the Catalonian sister programme ‘Urgències VIHgila’ (Watch out, EDs), were launched in 2021 to promote the implementation of these recommendations and the linkage of newly diagnosed patients to the healthcare system. Initial results have shown that these programmes are an effective strategy for diagnosing occult infections in the ED and also promote an increment of HIV screening in EDs for other clinical scenarios [21]. However, the demographic, clinical, microbiological and follow-up data of patients newly diagnosed with HIV infection with this opt-in strategy has yet to be described.

This study aims to evaluate the effectiveness of the opportunistic HIV screening programme in EDs in Catalonia, Spain, by assessing the number and proportion of new HIV diagnoses made within the six defined high-risk clinical scenarios. It also aims to describe the demographic and clinical profile of newly diagnosed patients, the clinical and laboratory characteristics identifying patients with recent or advanced infection and evaluate and identify differences in the rate of linkage to HIV care and initiation of antiretroviral treatment.

## Methods

### Study design

This was a descriptive, multicentre, retrospective study of the epidemiological, clinical, analytical and linkage to care characteristics of new HIV diagnoses performed in Catalonian EDs following the implementation of the *‘*Urgències VIHgila’ programme. This programme consists of an educational project addressed to ED physicians and nurses to increase HIV screening in EDs, especially in patients who fall into one or more of the six clinical scenarios recommended in the SEMES and GESIDA consensus document [20]. The programme is based on offering HIV testing to all patients who attended a participating ED with a condition potentially related to HIV infection (mainly associated with the six clinical scenarios described in the consensus document) or on the basis of the medical professional's judgement that the patient presented a clinical situation that could be associated with hidden HIV infection. All patients, after giving their informed consent, underwent HIV serological testing. On some occasions, the decision to perform HIV serological testing was influenced by the patient's final diagnosis, so some patients required a specific blood sample to be taken at a later date for HIV serological testing. The programme began in 10 Catalonian EDs in July 2021 and had expanded to 24 EDs by July 2024. A detailed description of the *‘*Urgències VIHgila’ programme has been previously published [22,23].

Emergency departments involved in the *‘*Urgències VIHgila’ programme for at least 2 years were invited to review all newly diagnosed individuals from the time they joined the project until March 2024, when the retrospective review planned in this study began. Individuals in whom serological confirmation or VL excluded HIV infection were not included and neither were individuals with previously known HIV infection linked to and receiving adequate care.

### Measurements

The diagnosis of HIV infection was via a positive fourth-generation serological test (antibodies against HIV-1/2 and p24 HIV-1 antigen) followed by a confirmatory serological HIV test and/or positive HIV VL. As this was a screening programme for occult HIV infection, the window period was not considered and a negative serology precluded VL testing unless there was a suspicion of acute HIV infection.

The main epidemiological characteristics of each person were collected: age, sex assigned at birth, sexual identity, sexual orientation and country of origin. Sexual orientation was defined as heterosexual for people having sex with people with a gender other than their own, non-heterosexual for people having sex with people of their same gender, bisexual people, and others. We also reported whether the patients were diagnosed in an ED in an urban (population of 200,000 or more) or rural (less than 200,000) area. Clinical variables related to the reason for requesting an HIV test, according to the consensus document, were also collected: previous HIV screening, suspected mode of infection, need for hospitalisation, time to referral to HIV clinic and initiation of ART [20]. Characteristics of PWH not linked to care were collected if reported in previous clinical records.

Laboratory data on Fiebig stage (acute infection defined by stages I to V and chronic infection by stage VI [24]), CD4 lymphocyte count (cells/mm^3^), CD4 lymphocyte percentage and HIV VL (copies/mL) were collected [25].

### Statistical analysis

All continuous quantitative variables were tested for normal distribution with a Shapiro-Wilk test. Normally distributed variables were compared using the Student's t-test and expressed as mean ± standard deviation. Variables with a non-normal distribution were analysed using the Mann–Whitney U test and expressed as median and interquartile range (IQR). Results were compared between groups stratified according to demographic and clinical variables, specifically sexual orientation (heterosexual or non-heterosexual), acute HIV infection or chronic infection and correctly linked or unlinked patient. Categorical variables were compared using the chi-square test or Fisher's exact test, as appropriate, and continuous variables were analysed using t-tests or non-parametric equivalents. A p value < 0.05 was considered statistically significant. For variables with missing data, a complete case analysis was performed on the total dataset without imputing values to obtain unbiased estimates. Data were analysed using Stata version 14 (StataCorp, College Station, United States (US)).

## Results

Seventeen EDs of the 24 EDs included in ‘Urgències VIHgila’ programme participated in the study. These 17 EDs provide healthcare to a catchment area of 5.9 million people (representing ca 73% of the 8 million inhabitants of Catalonia). These EDs participated in the *‘*Urgències VIHgila’ for a total of 489 months. Eleven EDs provided data from the beginning of the programme in July 2021 for a total of 33 months per ED. Initially only 10 EDs started the programme. However another ED joined 1 year later (July 2022) but was able to provide retrospective data from the start of the programme, as it had already begun implementing the same screening criteria before its official inclusion. Therefore, data from this ED were included from the beginning of the study. The remaining six EDs joined 1 year later (July 2022) for a participation of 21 months. During this period, 23,105 HIV serologies were performed in the participating EDs and a total of 172 people were newly diagnosed with HIV infection, obtaining a positivity rate of 0.7%.

Regarding the characteristics of newly diagnosed HIV-infected individuals, 152 (88.4%) were assigned male at birth and the median age of the entire sample was 39 years (IQR: 30–50 years). Among the 169 new diagnoses of HIV in individuals in whom their country of origin was known (98.3%), 81 (47.9%) were from Spain, 50 (29.6%) from Latin America, 16 (9.5%) from other European countries and 11 (6.5%) from Africa. Previous HIV screening information was available for 121 individuals (70.3% of the whole sample), of whom only 51 (42.1%) had been screened for HIV within the past 10 years. The most common mode of transmission documented was sexual transmission, observed in 130 cases (75.6%), although in up to 20.9% of cases, the source of infection was undetermined. In terms of sexual orientation, 81 (55.5%) defined themselves as heterosexual. The remaining sociodemographic characteristics are shown in Table 1.

**Table 1:** Sociodemographic, clinical and follow-up data of patients diagnosed with HIV at participating emergency departments, Catalonia, Spain, July 2021–March 2024 (n = 172 individuals)

Data	Total (n = 172)				
**Sociodemographic data**	**Numerator**	**Denominator**	**%**	**Median**	**IQR**
Assigned male at birth	152	172	88.4	NA	
Assigned female at birth	20	172	11.6	NA	
Sexual identity	160	172	93.0	NA	
Cisgender man	134	172	83.8	NA	
Cisgender woman	18	172	11.3	NA	
Transgender man	1	172	0.6	NA	
Transgender woman	7	172	4.1	NA	
Age (years)	172	NA	NA	39	30–50
Origin	169	172	98.3	NA	
European	97	169	57.4	NA	
Spain	81	169	47.9	NA	
Other European countries^a^	16	169	9.5	NA	
Non-European countries	72	169	42.6	NA	
Latin America^b^	50	169	29.6	NA	
Africa^c^	11	169	6.5	NA	
Others^d^	11	169	6.5	NA	
Previously screened	51	121	42.1	NA	
Urban area (over 200,000 inhabitants)	111	172	64.5	NA	
Infection transmission mechanism	172	172	100	NA	
Sexually transmitted	130	172	75.6	NA	
Intravenous drug use	6	172	3.5	NA	
Unknown	36	172	20.9	NA	
Sexual orientation	146	171	84.9	NA	
Heterosexual	81	146	55.5	NA	
Non-heterosexual	65	146	44.5	NA	
MSM	45	146	30.8	NA	
WSW	0	146	0	NA	
Bisexual	16	146	11.0	NA	
Others	4	146	2.7	NA	
**Clinical data**					
Reason for HIV screening	172	172	100	NA	
Conditions targeted by consensus document recommendations	94	172	54.6	NA	
Community-acquired pneumonia^e^	44	172	25.6	NA	
Sexually transmitted infection	21	172	12.2	NA	
Mononucleosis syndrome	12	172	7.0	NA	
HIV post-exposure prophylaxis	7	172	4.1	NA	
Herpes zoster^e^	7	172	4.1	NA	
Practicing chemsex	3	172	1.7	NA	
Other conditions	78	172	45.4	NA	
Fever of unknown origin^f^	18	78	23.1	NA	
Confusion/behavioural disturbances	7	78	9.0	NA	
Acute HIV infection (Fiebig stages I to V)	78	135	57.8	NA	
Viral load (copies/mL)	152	NA	NA	419,839	46,489–1,262,877
CD4^+^ lymphocytes (cells/mm^3^)	150	NA	NA	224	48–373
CD4^+^ lymphocytes under 200 cells/mm^3^	70	150	46.7	NA	
Advanced HIV infection (Fiebig stage VI and < 200 CD4^+^ lymphocytes)	30	124	24.2	NA	
**Follow-up data after ED care**					
Need for hospitalisation	88	172	51.2	NA	
Death during the index episode	3	172	1.7	NA	
Linkage to care after discharge (n = 169)^e^	137	167	82.0	NA	
Days from diagnosis to first outpatient visit (n = 169)^g^	142	NA	NA	8	3–15
Days from diagnosis to start antiretroviral treatment (n = 169)^g^	142	NA	NA)	9	4–17

IQR: interquartile range; MSM: men who have sex with men; NA: not applicable; WSW: women who have sex with women.

^a^ Newly diagnosed patients in this study originated from Andorra, Belgium, Denmark, Italy, Latvia, Poland, Romania and Ukraine.

^b^ Newly diagnosed patients in this study originated from Argentina, Bolivia, Brazil, Chile, Colombia, Costa Rica, Cuba, Ecuador, Honduras, Mexico, Paraguay, Peru, Dominican Republic and Venezuela.

^c^ Newly diagnosed patients in this study originated from Burkina Faso, Cameroon, Gambia, Ghana, Guinea-Bissau, Equatorial Guinea, Mali, Morocco and Senegal.

^d^ Newly diagnosed patients in this study originated from Bangladesh, India, Pakistan, Russia and Singapore.

^e^ The three patients who died during the index episode were not considered.

^f^ A fever was defined as a temperature above 38 °C as recorded by a healthcare professional.

^g^ Only individuals who linkage to care were considered.

On looking at the clinical data of the HIV infections, 94 (54.6%) of the 172 HIV diagnoses were related to the six clinical scenarios recommended in the consensus document for HIV screening in EDs [20]. The most frequent diagnoses from these six clinical scenarios were CAP in 44 (25.6%) cases and STIs in 21 (12.2%) cases. For the remaining HIV testing requests outside the consensus document recommendations, the most common reasons for screening were fever of unknown origin (n = 18, 23.1%) and confusion or behavioural disturbances (n = 7, 9.0%). Microbiological laboratory data of Fiebig stages were available for 135 (78.5%) of the 172 patients diagnosed with HIV in the ED. According to the Fiebig stage, 78 (57.8%) of these 135 individuals had acute HIV infection (Fiebig stages I to V) with a median age of 38 years (IQR: 29–45 years), a median VL of 706,953 copies/mL (IQR: 84,282–330,9125 copies/mL) and a median CD4^+^ cell count of 300 cells/mm^3^ (IQR: 64–360 cells/mm^3^). Thirty PWH (24.2%) were diagnosed with an advanced HIV stage, as they were in Fiebig stage VI and had less than 200 CD4^+^/mm^3^. The remaining clinical and laboratory observations are shown in Table 1.

With respect to follow-up data after ED care of patients with newly diagnosed HIV infection, 88 patients (51.2%) required hospitalisation and three died during their hospital stay (in-hospital mortality: 1.7%). Of the three patients who died, one was a cisgender heterosexual male in their mid-40s, with no prior screening or identification of factors associated with HIV infection, who died of severe pneumonia. The second was a cisgender female in their 60s, with no identified risk factors and no previous HIV screening, who was admitted for multi-organ failure associated with acute hepatitis. The third was a Latin-American cisgender homosexual male around 50 years old, with no information about previous screening, who was admitted with fever and died of septic shock. These three patients’ CD4^+^ count at the time of HIV diagnosis was 20, unknown and 100 cells/mm^3^, respectively.

Despite all the patients being referred to outpatient HIV clinics after the diagnosis of HIV, linkage to care was effectively implemented in 136 (80.5%) of the 169 individuals diagnosed with HIV in the ED and discharged alive, with the remaining 32 (19.0%) being lost to follow up. The median time from diagnosis to the first HIV specialist care visit was 8 days (IQR: 3–15 days) and the median time to initiation of ART was 9 days (IQR: 4–17 days), regardless of whether the individual was admitted or discharged directly from the ED. Social reasons (being a foreigner, change of residence, lack of contact data) or simply not attending consultations after discharge accounted for most of the 32 PWH who were not linked to care. The complete follow-up data are shown in Table 1.

### Heterosexual versus non-heterosexual people with HIV

In relation to individual characteristics, a specific comparison was also made between heterosexual and non-heterosexual PWH (Table 2). On comparing the heterosexual group with the non-heterosexual group, there was a significantly lower representation of heterosexual patients among those diagnosed in EDs in rural areas (45.7% vs 29.2%, p = 0.03), a lower proportion of infection associated with sexual transmission (65.4% vs 95.4%, p < 0.01) and fewer heterosexual patients had been previously screened for HIV (25.4% vs 41.5%, p < 0.01). Acute HIV infections were detected in 67.7% of heterosexual individuals vs in 41.5% of non-heterosexual individuals, although this difference did not reach statistical significance (p = 0.09). However, there were significant differences in VL levels, which were higher in the heterosexual group (Table 2), consistent with the findings of a higher number of acute infections in this group. A greater need for hospitalisation was reported in heterosexual group (59.7% vs 40%, p = 0.02) and this group had a higher level of adherence to the first visit than non-heterosexuals (87.3% vs 75%, p = 0.05).

**Table 2:** Comparison of heterosexual and non-heterosexual patients diagnosed with HIV at participating emergency departments, Catalonia, Spain, July 2021–March 2024 (sexual orientation data available for n = 146 individuals)

Characteristics	Heterosexual orientation (n = 81)					Non-heterosexual orientation (n = 65)					p value
	Numerator	Denominator	%	Median	IQR	Numerator	Denominator	%	Median	IQR	
Assigned male at birth	65	81	80.2	NA		63	65	96.9	NA		**0.01**
Assigned female at birth	16	81	19.8	NA		2	65	3.1	NA		
Age (years)	81	NA	NA	40	31–51	65	NA	NA	39	29–49	0.4
European origin	49	81	61.3	NA		37	65	57.0	NA		0.4
Previously screened	14	81	25.4	NA		27	65	41.5	NA		**< 0.01**
Urban area (over 200,000 inhabitants)	44	81	54.3	NA		46	65	70.8	NA		**0.03**
Sexually transmitted	53	81	65.4	NA		62	65	95.4	NA		**< 0.01**
HIV screening triggered by one of the six clinical scenarios recommended in the consensus document	35	81	43.2	NA		42	65	64.6	NA		**< 0.01**
Acute HIV infection (Fiebig stages I to V)	42	81	67.7	NA		27	65	41.5	NA		0.09
Viral load (copies/mL)	81	NA	NA	702,801	94,000–1,806,212	65	NA	NA	103,901	16,321–538,208	**< 0.01**
CD4^+^ lymphocytes (cells/mm^3^)	81	NA	NA	170	40–360	65	NA	NA	284	68–452	0.1
CD4^+^ under 200 cells/mm^3^	36	81	44.4	NA		24	65	36.9	NA		0.4
Advanced HIV infection (Fiebig stage VI and CD4^+^ < 200 cells/mm^3^)^a^	11	81	19.0	NA		13	65	27.1	NA		0.3
Need of hospitalisation	46	81	59.7	NA		26	65	40.0	NA		**0.02**
Linkage to care^b^	69	79	87.3	NA		48	64	75	NA		**0.05**
Days from diagnosis to first outpatient visit^c^	79	NA	NA	10	4–17	64	NA	NA	6	2–13	0.1
Days from diagnosis to start antiretroviral treatment^c^	79	NA	NA	11	5–21	64	NA	NA	8	4–19	0.2

IQR: interquartile range; NA: not applicable.

^a^ Percentage was calculated on the total number of individuals for whom Fiebig stage and CD4 count were available (n = 124).

^b^ The three patients who died during the index episode were not considered.

^c^ Only individuals with linkage to care were considered.

Statistically significant differences are indicated in bold.

For those with missing data, a full analysis of cases on the total without imputation of values was performed to obtain unbiased estimates.

The expanded data on sexual identities appear in Supplementary Table S1.

### Newly diagnosed HIV-infected individuals

Table 3 shows a comparison of newly diagnosed HIV-infected patients according to their HIV infection stage (Fiebig classification) at diagnosis. Individuals with acute HIV infection (Fiebig stages I to V) were younger than those with chronic infection (Fiebig stage VI), with median age of 38 vs 41 years (p = 0.03). Individuals diagnosed with chronic infection were more frequently of non-European origin and living in urban areas, whereas those diagnosed with acute infection were more frequently of European origin, mainly Spanish, and living in rural areas (p = 0.03 and p < 0.01, respectively). Individuals with acute HIV infection had a higher VL and higher CD4^+^ cell count than those without acute infection, both statistically significant (p = 0.02 and p = 0.01, respectively). Of the 124 individuals for whom Fiebig stage and CD4^+^ count were available, 30 had advanced infection (Fiebig stage VI and CD4+ count below 200 cells/mm^3^), representing 24.2% of new HIV diagnoses made in the ED.

**Table 3:** Characteristics of patients with acute or chronic HIV infection according to the Fiebig stage classification, diagnosed at participating emergency departments, Catalonia, Spain, July 2021–March 2024 (Fiebig stage data available for n = 135 individuals)

Characteristics	Acute HIV infection (Fiebig stages I to V) (n = 78)					Chronic HIV infection (Fiebig stage VI) (n = 57)					p value
	Numerator	Denominator	%	Median	IQR	Numerator	Denominator	%	Median	IQR	
Assigned male at birth	69	78	88.5	NA		52	57	91.2	NA		0.4
Assigned female at birth	9	78	11.5	NA		5	57	8.8	NA		
Age (years)	78	NA	NA	38	29–45	57	NA	NA	41	33–54	**0.03**
European origin	48	78	62.3	NA		25	57	43.9	NA		**0.03**
Previously screened	25	78	32.1	NA		18	57	31.6	NA		0.5
Urban area (over 200,000 inhabitants)	52	78	66.7	NA		52	57	91.2	NA		**< 0.01**
Sexually transmitted	57	78	73.1	NA		46	57	80.7	NA		0.4
Heterosexual orientation	42	78	60.9	NA		20	57	44.4	NA		0.06
HIV screening triggered by one of the six clinical scenarios recommended in the consensus document	38	78	48.7	NA		36	57	63.2	NA		0.1
Viral load (copies/mL)	78	NA	NA	706,953	84,282–3,309,125	57	NA	NA	214,263	4,886– 875,976	**0.02**
CD4^+^ lymphocytes (cells/mm^3^)	78	NA	NA	300	64–453	57	NA	NA	148	30–308	**0.01**
CD4^+^ under 200 cells/mm^3^	28	78	36.0	NA		30	57	52.6	NA		**0.04**
Need of hospitalisation	39	78	52.7	NA		34	57	60.7	NA		0.4
Linkage to care^a^	67	78	88.2	NA		44	57	77.2	NA		0.3
Days from diagnosis to first outpatient visit^b^	76	NA	NA	8	5–14	56	NA	NA	9	3–16	0.8
Days from diagnosis to start antiretroviral treatment^b^	76	NA	NA	8	5–16	56	NA	NA	9	5–16	0.8

IQR: interquartile range; NA: not applicable.

^a^ The three patients who died during the index episode were not considered.

^b^ Only individuals with linkage to care were considered.

Statistically significant differences are indicated in bold.

For variables with missing data, a full analysis of cases on the total without imputation of values was performed to obtain unbiased estimates.

The expanded data on sexual identities appear in Supplementary Table S2.

### Linkage to HIV specialist care

A higher rate of linkage to HIV specialist care was reported for those with acute infection than those with chronic infection (89.3% vs 81.5%, respectively, p = 0.3). Aside from a relationship between acute infection and heterosexual orientation, there were no additional factors associated with successful linkage to care (Table 4). The six clinical scenarios targeted by the recommendations of the consensus document tended to be associated with a higher diagnosis of chronic infections, but not statistically significant (p = 0.1).

**Table 4:** Comparison of patients diagnosed with HIV at participating emergency departments with successful and unsuccessful linkage to care in outpatient HIV clinics, Catalonia, Spain, July 2021–March 2024 (linkage data available for n = 164 individuals)

Characteristics	Successful linkage to care (n = 136)					Unsuccessful linkage to care (n = 28)					p value
	Numerator	Denominator	%	Median	IQR	Numerator	Denominator	%	Median	IQR	
Assigned male at birth	121	136	89.0	NA		24	28	85.7	NA		0.4
Assigned female at birth	15	136	11	NA		4	28	14.3	NA		
Age (years)	136	136	NA	39	31–49	28	NA	NA	37	28–49	0.3
European origin	77	136	56.6	NA		16	28	61.5	NA		0.4
Previously screened	42	NA	30.9	NA		8	28	28.6	NA		0.9
Urban area (over 200,000 inhabitants)	90	136	65.7	NA		17	28	56.7	NA		0.4
Sexually transmitted	107	136	78.7	NA		20	28	71.4	NA		0.5
Heterosexual orientation	69	136	59.0	NA		9	28	37.5	NA		***0.05***
HIV screening triggered by one of the six clinical scenarios recommended in the consensus document	76	136	55.9	NA		14	28	50.0	NA		0.7
Acute HIV infection (Fiebig I–V)	67	136	60.4	NA		8	28	44.4	NA		0.3
Viral load (copies/mL)	136	NA	NA	419,839	48,860–1,130,000	28	NA	NA	317,315	65,300–2,760,000	0.7
CD4 lymphocytes (cells/mm^3^)	136	NA	NA	234	48–386	28	28	NA	206	118–308	0.9
CD4^+^ under 200 cells/mm^3^	61	136	46.2	NA		7	28	46.7	NA		0.6
Advanced HIV infection (Fiebig stage VI and CD4^+^ < 200 cells/mm^3^)^a^	25	136	22.9	NA		4	28	33.3	NA		0.4
Need of hospitalisation	75	136	56.0	NA		11	28	40.7	NA		0.2
Days from diagnosis to first outpatient visit	136	NA	NA	8	3–15	28	NA	NA	NA		NA
Days from diagnosis to start antiretroviral treatment	136	NA	NA	9	5–17	28	NA	NA	NA		NA

IQR: interquartile range; NA: not applicable.

^a^ Percentage was calculated on the total number of individuals for whom Fiebig stage and CD4 count were available (n = 124).

Statistically significant differences are indicated in bold.

For variables with missing data, a full analysis of cases on the total without imputation of values was performed to obtain unbiased estimates.

The expanded data on sexual identities appear in Supplementary Table S3.

## Discussion

Testing for HIV infection is a crucial preventive measure, which has led many countries to involve EDs in efforts to diagnose occult infections. In 2006, the US Centers for Disease Control and Prevention (CDC) updated the US guidelines for HIV testing in healthcare settings, recommending widespread non-targeted opt-out HIV screening in settings in which the prevalence of undiagnosed infection was 0.1% or higher [26]. In 2021, the United Kingdom government’s Zero HIV Action Plan advocated for opt-out HIV testing in high-prevalence areas. A 9-month screening programme in 33 EDs yielded 3,664 positive results among 665,746 tests, including 282 (7.7%) new diagnoses and 144 (3.9%) people who were lost to care [16]. Despite a new diagnosis rate of 0.04% (0.06% if individuals not receiving care were included), which was lower than the 0.1% cut-off for cost-effectiveness, the opt-out testing programme was deemed a successful public health strategy [27]. However, the possibility to implement a universal opt-out HIV testing strategy in Spain is limited due to the requirement of explicit consent for HIV testing. Spanish guidelines for HIV screening in healthcare settings (updated in 2014) recommend HIV-targeted opt-in testing for PWH or AIDS-defining clinical conditions and people with suspected HIV risk factors, apart from routine and mandatory situations [28]. Despite these recommendations, 2,956 new HIV diagnoses were registered in Spain in 2022 (around 7.7 cases per 100,000 inhabitants, higher than the mean of other European Union countries), with a relevant proportion (48.6%) in late and advanced stages [5].

The opt-in strategy developed by the ‘Urgències VIHgila’ programme to promote HIV screening in EDs in Catalonia has achieved a positivity rate of 0.7% [29]. This is more than twice the estimated prevalence of 0.31% for the Spanish population in the 2022 report [5]. This increase was probably due to a positive selection phenomenon given the conditions established for screening as well as the sensitisation of emergency physicians, thus demonstrating this strategy as effective. The focus was on the six clinical scenarios; however, the implementation of the programme increased general HIV awareness in EDs, which has led to increased HIV testing in other clinical situations [22]. In our cohort, 45.4% of the 172 new HIV diagnoses were from testing requested outside the consensus document recommendations, particularly in people with fever of unknown origin, confusion or behavioural disturbances and people using various types of drugs (not limited to using drugs intravenously) [30,31]. The HIV testing requested outside the consensus document recommendations is probably due to increased awareness among health professionals, which has led to the implementation of an HIV screening programme in EDs. The programme allows for requests outside the six clinical scenarios in order to keep the programme dynamic, with constant reevaluation to assess the expansion to new settings and reassess proposals to improve the effectiveness of the programme. Future analysis should explore whether the criteria for HIV screening in the ED, supported by the consensus document, should be extended. Our findings underscore the important role of EDs in the diagnosis of occult HIV infection and in reducing late diagnoses.

Regarding the demographic characteristics of the individuals newly diagnosed with HIV in our cohort, we found some differences with respect to the annual Spanish and Catalonian reports for 2022. First, it should be noted that most of the people included in our study were classified as male at birth (83%). There are several reasons for this. Many of the large hospitals involved in the ‘Urgències VIHgila’ project have a separate emergency gynaecological unit. Since STIs are one of the conditions detected in HIV screening tests, and the project is only conducted in the general emergency department, it is possible that women are not being screened when they present with suspected STIs. In addition, women have often been perceived as being at lower risk of STIs, including HIV, which may have contributed to them being offered testing less frequently - a pattern suggested by recent data from Catalan EDs (data not shown). This reveals a gender bias that opens the door for future studies in this area. Second, concerning participant origin, our results were similar to the Spanish 2022 report (47.9% Spanish and 29.6% Latin American origin in our study vs 51.5% and 31.5% in the Spanish report, respectively) [8], but different from those in the Catalonian 2022 report, in which 40.8% were Spanish and 39.3% were from Latin America [9]. We also found differences in the most frequent mode of transmission. While 55% and 58.8% of new HIV diagnoses were reported to be in MSM in Spain and Catalonia, respectively, they only constituted 30.8% in our cohort, with 55.5% of new cases being sexually transmitted through heterosexual intercourse [8,9]. These facts are probably explained by the existence of many community-based strategies and points of care for HIV diagnosis in Catalonia for specific groups, such as migrants, sex workers or non-heterosexual people, which are not frequented by the cis-heterosexual community. In addition, heterosexual individuals are still less aware of their vulnerability to HIV infection [3,32,33]. These reasons probably justify opportunistic screening in this population in the ED and outside the previously existing usual circuits. Thus, ED testing could have a very high impact in uncovering HIV infection in individuals with heterosexual orientation.

Up to 57.8% of HIV infections detected in our cohort in the participating EDs were in an acute phase of infection according to the Fiebig classification. This is important because it detects people in their most infectious phase, allowing early treatment to begin and thereby greatly reducing community transmission. As expected, patients with acute infection had higher VL and CD4^+^ cell counts than those with chronic infection. The detection of up to 58% of individuals in the acute phase with a median VL greater than 700,000 copies/mL is a key public health feature of an occult infection detection programme such as ‘Urgències VIHgila’ as this profile of individuals has the highest risk of transmission due to their high VL. This fact confirms ED screening as a highly valuable programme within HIV public health strategies. On the other hand, it was observed that more than 20% of people were diagnosed with advanced HIV, allowing their linkage to care and initiation of ART, potentially preventing progression to opportunistic infections and other AIDS-related complications. Community-acquired pneumonia was the clinical scenario associated with the highest percentage of HIV diagnoses (25.6%), which highlights the importance of this scenario within the suspicion of occult HIV infection, mainly in people between 18 and 65 years of age. Despite this fact, HIV testing is still dismissed in EDs in 4 of 5 patients with CAP [22]. The clinical scenario associated with the second highest percentage of HIV diagnoses was STIs in 12.2% of cases. These facts justify the need to integrate the diagnosis and treatment of CAP and STIs with HIV screening in emergency medical care, and every campaign to improve rates of screening will positively serve to increase detection of HIV in EDs.

Once an HIV diagnosis has been made, efficient linkage with an infectious diseases ambulatory consultation is necessary to achieve the second and third 95 promoted by UNAIDS. In this regard, the *‘*Urgències VIHgila’ programme encouraged health centres to create more efficient circuits among EDs, microbiologists and infectious disease clinicians. In our cohort, we observed that the programme enabled most people newly diagnosed with HIV to be referred to a specialist and start ART within 1 week; however, this time was longer if the person remained hospitalised or had a pathology which deferred ART initiation. Overall, we found high linkage to care rates of around 80% and, remarkably, ca 90% of acutely infected participants being linked to ambulatory care within a short period of time after ED care and diagnosis. Nonetheless, these figures are far from the overall 96.6% linkage to care reported in Spain. Factors that may have influenced this (such as migration, distance to hospital, co-morbidity, social support, HIV-related stigma, mental illness, substance abuse and misuse, among others) were not specifically analysed as most of them are not recorded in clinical records as part of care. This fact indicates the need to target efforts to prioritise certain vulnerable populations and to adapt resources and diagnosis and linkage efforts. In cases in which social and structural barriers hinder linkage to care and retention in HIV care (and general healthcare), EDs have an important role in supporting people with a previous HIV diagnosis and no linkage to care by trying to direct them to specialised consultation. It is key to increase the percentage of patients diagnosed with HIV in EDs who are finally and effectively linked to outpatient care.

As this was a retrospective study, one limitation is that we were unable to obtain all the planned data for all participants, and some biased estimations may have been present in our findings. Second, we did not analyse the missed opportunities of the new diagnoses; however, the fact that only 29.7% of individuals with previous HIV screening information available in their medical report had been screened for HIV within the past 10 years highlights the urgent need to bring HIV testing closer to the population. Third, the relatively low number of cases detected implies that a low statistical power and a beta-error in some comparisons could exist in our estimations. Finally, HIV new diagnoses were defined when a previous positive HIV test has not been identified in the system or the diagnosis did not appear in previous medical reports, which can lead to a biased estimation of the positivity rate.

## Conclusions

Our study demonstrates the crucial role of EDs in the diagnosis of occult HIV infections. It shows that the targeted opt-in strategy promoted by the ‘Urgències VIHgila*’* programme to test for HIV in patients coming to the Catalonian EDs is an effective strategy, having rendered a rate of 0.7% of positive tests. Second, it defined the characteristics of the 172 newly diagnosed HIV-infected individuals, which are largely different from those reported for patients diagnosed at other healthcare levels. Third, it revealed that more than half of the new diagnoses corresponded to individuals with acute infection and nearly a quarter had late-stage HIV. And fourth, despite the effort made to uncover previously unknown HIV infections, one in five patients were not effectively linked to an outpatient HIV clinic. Consequently, it can be postulated that EDs are emerging as new frontline advocates in the fight against the HIV epidemic, contributing considerably to progress towards the UNAIDS targets and ultimately bringing the HIV epidemic closer to an end. Greater focus and effort on linkage to care strategies could yield even better results.

## Data Availability

All data generated or analysed during this study are included in this published article.

## Supplementary Data

290000 Supplementary Material

## References

[1] World Health Organization (WHO). HIV data and statistics. Geneva; WHO. [Accessed: 5 Apr 2025]. Available from: https://www.who.int/teams/global-hiv-hepatitis-and-stis-programmes/hiv/strategic-information/hiv-data-and-statistics

[2] Agustí C Montoliu A Mascort J Carrillo R Almeda J Elorza JM Missed opportunities for HIV testing of patients diagnosed with an indicator condition in primary care in Catalonia, Spain. Sex Transm Infect. 2016;92(5):387-92. 10.1136/sextrans-2015-052328 26888659

[3] Youssef E Wright J Delpech V Davies K Brown A Cooper V Factors associated with testing for HIV in people aged ≥50 years: a qualitative study. BMC Public Health. 2018;18(1):1204. 10.1186/s12889-018-6118-x 30367609 PMC6204048

[4] Rava M Domínguez-Domínguez L Bisbal O López-Cortés LF Busca C Antela A Late presentation for HIV remains a major health issue in Spain: Results from a multicenter cohort study, 2004-2018. PLoS One. 2021;16(4):e0249864. 10.1371/journal.pone.0249864 33882093 PMC8059864

[5] Moreno S Berenguer J Fuster-Ruizdeapodaca MJ García Ontiveros M. Detección temprana. Enferm Infecc Microbiol Clin. 2018;36:35-9. 10.1016/S0213-005X(18)30245-3 30115407

[6] Marks G Crepaz N Senterfitt JW Janssen RS. Meta-analysis of high-risk sexual behavior in persons aware and unaware they are infected with HIV in the United States: implications for HIV prevention programs. J Acquir Immune Defic Syndr. 2005;39(4):446-53. 10.1097/01.qai.0000151079.33935.79 16010168

[7] Marks G Crepaz N Janssen RS. Estimating sexual transmission of HIV from persons aware and unaware that they are infected with the virus in the USA. AIDS. 2006;20(10):1447-50. 10.1097/01.aids.0000233579.79714.8d 16791020

[8] National Centre for Epidemiology, Institute of Health Carlos III (ISCIII-CNE). Vigilancia epidemiológica del VIH y el SIDA en España, 2022. Sistema de información sobre nuevos diagnósticos de VIH y registro nacional de casos de SIDA. [HIV and AIDS epidemiological surveillance in Spain, 2022: New HIV diagnoses information system and national AIDS case registry]. Madrid: ISCIII-CNE; 2023. Spanish. Available from: sanidad.gob.es/ciudadanos/enfLesiones/enfTransmisibles/sida/vigilancia/docs/Informe_VIH_SIDA_2023.pdf

[9] Centre d’Estudis Epidemiològics sobre les Infeccions de Transmissió Sexual i Sida de Catalunya (CEEISCAT). Vigilància Epidemiològica de la infecció pel VIH i la SIDA a Barcelona. Informe anual 2022. [HIV and AIDS epidemiological surveillance in Catalonia. Annual report 2022]. Badalona: CEEISCAT; 2023. Catalan. Available from: aspb.cat/wp-content/uploads/2023/11/ASPB-vigilancia-vih-sida-barcelona-2022-30112023.pdf

[10] Joint United Nations Programme on HIV/AIDS (UNAIDS). Understanding Fast-Track: accelerating action to end the AIDS epidemic by 2030. Geneva: UNAIDS; 2015. [Accessed: 10 January 2021]. Available from: unaids.org/sites/default/files/media_asset/201506_JC2743_Understanding_FastTrack_en.pdf

[11] National Centre for Epidemiology, Institute of Health Carlos III (ISCIII-CNE). Actualización del Continuo de Atención del VIH en España, 2021. [Update of the HIV Continuum of Care in Spain, 2021]. Madrid: ISCIII-CNE; 2023. Spanish. Available from: sanidad.gob.es/ciudadanos/enfLesiones/enfTransmisibles/sida/vigilancia/docs/Continuo_atencion_VIH_mayo_2023.pdf

[12] González Del Castillo J. Papel actual de los servicios de urgencias hospitalarios en la lucha contra la pandemia VIH. [Current role of hospital emergency departments in the fight against the human immunodeficiency virus infection pandemic]. Emergencias. 2021;33(1):7-8. 33496393

[13] Valero-Verdejo L Hueso-Montoro C Pérez-Morente MÁ. Evaluation of HIV screening in hospital emergency services. Systematic review. Int Emerg Nurs. 2023;71:101355. 10.1016/j.ienj.2023.101355 37852058

[14] Gillet C Darling KEA Senn N Cavassini M Hugli O. Targeted versus non-targeted HIV testing offered via electronic questionnaire in a Swiss emergency department: A randomized controlled study. PLoS One. 2018;13(3):e0190767. 10.1371/journal.pone.0190767 29513659 PMC5841645

[15] Reyes-Urueña J Fernàndez-López L Force L Daza M Agustí C Casabona J. Estudio del impacto a nivel de salud pública del cribado universal del virus de la inmunodeficiencia humana en un servicio de Urgencias. [Level of impact on the public health of universal human immunodeficiency virus screening in an Emergency Department]. Enferm Infecc Microbiol Clin. 2017;35(7):434-7. 10.1016/j.eimc.2015.06.014 26341042

[16] The Lancet HIV. Opt-out HIV testing in the UK. Lancet HIV. 2023;10(6):e351. 10.1016/S2352-3018(23)00117-0 37270223

[17] Prabhu VS Farnham PG Hutchinson AB Soorapanth S Heffelfinger JD Golden MR Cost-effectiveness of HIV screening in STD clinics, emergency departments, and inpatient units: a model-based analysis. PLoS One. 2011;6(5):e19936. 10.1371/journal.pone.0019936 21625489 PMC3098845

[18] Paltiel AD Weinstein MC Kimmel AD Seage GR 3rd Losina E Zhang H Expanded screening for HIV in the United States--an analysis of cost-effectiveness. N Engl J Med. 2005;352(6):586-95. 10.1056/NEJMsa042088 15703423

[19] Bauman LJ Braunstein S Calderon Y Chhabra R Cutler B Leider J Barriers and facilitators of linkage to HIV primary care in New York City. J Acquir Immune Defic Syndr. 2013;64(01) Suppl 1;S20-6. 10.1056/NEJMsa042088 24126445 PMC3888646

[20] González Del Castillo J Burillo-Putze G Cabello A Curran A Jaloud Saavedra E Malchair P Recomendaciones dirigidas a los servicios de urgencias para el diagnóstico precoz de pacientes con sospecha de infección por VIH y su derivación para estudio y seguimiento. [Recommendations for the early diagnosis of suspected human immunodeficiency virus infection in the emergency department and the referral of patients for follow-up: a consensus statement of the Spanish Society of Emergency Medicine (SEMES)]. Emergencias. 2020;32(6):416-26. 33275363

[21] González Del Castillo J Mirò E Miguens I Trenc P Espinosa B Piedrafita L Feasibility of a selective targeted strategy of HIV testing in emergency departments: a before-after study. [Emergency HIV network investigators. Feasibility of a selective targeted strategy of HIV testing in emergency departments: a before-after study]. Eur J Emerg Med. 2024;31(1):29-38. 10.1097/MEJ.0000000000001078 37729041

[22] Miró E Miró Ò Varón A Marrón P Canóniga C Salgado P Urgències VIHgila. Impacto de una formación específica y generalizada de los profesionales de enfermería en el despistaje en urgencias de infección oculta por VIH: experiencia del proyecto "Urgències VIHgila". Emergencias (Madr). 2024;36(3):188-96. 10.55633/s3me/019.2024 38818984

[23] Miró O Miró E Carbó M Saura M Rebollo A de Paz R [Emergency detection of HIV infection in patients consulting for conditions potentially related to occult infection: Initial results of the "Urgències VIHgila" program]. Rev Esp Quimioter. 2023;36(2):169-79. 10.37201/req/085.2022 36645021 PMC10066909

[24] Fiebig EW Wright DJ Rawal BD Garrett PE Schumacher RT Peddada L Dynamics of HIV viremia and antibody seroconversion in plasma donors: implications for diagnosis and staging of primary HIV infection. AIDS. 2003;17(13):1871-9. 10.1097/00002030-200309050-00005 12960819

[25] McMichael AJ Borrow P Tomaras GD Goonetilleke N Haynes BF. The immune response during acute HIV-1 infection: clues for vaccine development. Nat Rev Immunol. 2010;10(1):11-23. 10.1038/nri2674 20010788 PMC3119211

[26] Branson BM Handsfield HH Lampe MA Janssen RS Taylor AW Lyss SB Revised recommendations for HIV testing of adults, adolescents, and pregnant women in health-care settings. MMWR Recomm Rep. 2006;55(RR-14):1-17, quiz CE1-4. 16988643

[27] Sullivan AK Raben D Reekie J Rayment M Mocroft A Esser S Feasibility and effectiveness of indicator condition-guided testing for HIV: results from HIDES I (HIV indicator diseases across Europe study). PLoS One. 2013;8(1):e52845. 10.1371/journal.pone.0052845 23341910 PMC3546115

[28] Spanish Ministry of Health, Social Services and Equality. Guía de recomendaciones para el diagnóstico precoz de VIH en el ámbito sanitario, 2014. [Guidelines for early HIV diagnosis in healthcare settings]. Madrid: Spanish Ministry of Health; 2014. Spanish. Available from: sanidad.gob.es/ciudadanos/enfLesiones/enfTransmisibles/sida/docs/GuiaRecomendacionesDiagnosticoPrecozVIH.pdf

[29] Urgències VIHgila group and the Catalan Health Department. Informe del Programa Urgències VIHgila. [Urgències VIHgila programme report]. [Accessed: 22 Nov 2024]. Catalan. Available from: https://www.urgencies-vihgila.cat/

[30] Puiguriguer Ferrando J. Intoxicaciones y VIH: otra nueva oportunidad. [Poisoning and HIV: another new opportunity]. Emergencias. 2023;35(2):85-6. 37038936

[31] Losada A Supervía A Vallecillo G Petrus C Aranda D Chen J Intoxicaciones por drogas de abuso: características diferenciales en población VIH. [Patients with drug-abuse poisoning with and without HIV infection: differential characteristics]. Emergencias. 2023;35(2):103-8. 37038940

[32] Gwadz M Leonard NR Honig S Freeman R Kutnick A Ritchie AS. Doing battle with "the monster:" how high-risk heterosexuals experience and successfully manage HIV stigma as a barrier to HIV testing. Int J Equity Health. 2018;17(1):46. 10.1186/s12939-018-0761-9 29678188 PMC5910579

[33] Van der Bij AK Dukers NH Coutinho RA Fennema HS. Low HIV-testing rates and awareness of HIV infection among high-risk heterosexual STI clinic attendees in The Netherlands. Eur J Public Health. 2008;18(4):376-9. 10.1093/eurpub/ckm120 18381296

