# Supplementary material for: Characterisation of new HIV diagnoses achieved in emergency departments using an opt-in strategy, Catalonia, Spain, July 2021 to March 2024
Source: Euro Surveill. 2025 Nov 27;30(47):2400781. doi: 10.2807/1560-7917.ES.2025.30.47.2400781 (PMC12788720; doi:10.2807/1560-7917.ES.2025.30.47.2400781)
Supplement: Supplementary Material [file 24-00781_ALVAREZ-LOPEZ_Supplement.pdf]

This supplementary material is hosted by *Eurosurveillance* as supporting information alongside the article 'Characterisation of new HIV diagnoses achieved in emergency departments using an opt-in strategy, Catalonia, Spain, July 2021 to March 2024', on behalf of the authors, who remain responsible for the accuracy and appropriateness of the content. The same standards for ethics, copyright, attributions and permissions as for the article apply. Supplements are not edited by *Eurosurveillance* and the journal is not responsible for the maintenance of any links or email addresses provided therein.

Supplementary Table S1. Comparison of heterosexual and non-heterosexual patients diagnosed with HIV at participating emergency departments, Catalonia, Spain, July 2021–March 2024 (sexual orientation data available for n = 146 individuals). *A breakdown of the available data on sexual identity for the individuals being compared.*

Characteristics	Heterosexual orientation (n = 81)					Non-heterosexual orientation (n = 65)					P value
	Numerator	Denominator	%	Median	IQR	Numerator	Denominator	%	Median	IQR	
Assigned male at birth	65	81	80.2	NA		63	65	96.9	NA		***0.01***
Assigned female at birth	16	81	19.8	NA		2	65	3.1	NA		
Cisgender man	62	81	76.5	NA		59	65	90.8	NA		***0.02***
Cisgender woman	14	81	17.3	NA		2	65	3.1	NA		***0.01***
Transgender man	1	81	1.2	NA		0	65	0	NA		0.6
Transgender woman	3	81	3.7	NA		1	65	1.5	NA		0.4

Supplementary TABLE S2. Characteristics of patients with acute or chronic HIV infection according to the Fiebig stage classification, diagnosed at participating emergency departments, Catalonia, Spain, July 2021–March 2024 (Fiebig stage data available for n = 135 individuals). *A breakdown of the available data on sexual identity for the individuals being compared.*

Characteristics	Acute HIV infection (Fiebig stages I to V) (n = 78) Numerator	Denominator	%	Median	IQR	Chronic HIV infection (Fiebig stage VI) (n = 57) Numerator	Denominator	%	Median	IQR	p value
Assigned male at birth	69	78	88.5	NA		52	57	91.2	NA		0.4
Assigned female at birth	9	78	11.5	NA		5	57	8.8	NA		
Cisgender man	61	78	78.2	NA		47	57	82.5	NA		0.5
Cisgender woman	9	78	11.5	NA		5	57	8.8	NA		0.6
Transgender man	0	78	0	NA		0	57	0	NA		NA
Transgender woman	5	78	6.4	NA		2	57	3.5	NA		0.4

Supplementary Table S3. Comparison of patients diagnosed with HIV at participating emergency departments with successful and unsuccessful linkage to care in outpatient HIV clinics, Catalonia, Spain, July 2021–March 2024 (linkage data available for n = 164 individuals). *A breakdown of the available data on sexual identity for the individuals being compared.*

Characteristics	Successful linkage to care (n = 136)					Unsuccessful linkage to care (n = 28)					p value
	**Numerator**	**Denominator**	**%**	**Median**	**IQR**	**Numerator**	**Denominator**	**%**	**Median**	**IQR**	
Assigned male at birth	121	136	89.0	NA		24	28	85.7	NA		0.4
Assigned female at birth	15	136	11	NA		4	28	14.3	NA		
Cisgender man	108	136	79.4	NA		22	28	78.6	NA		0.9
Cisgender woman	13	136	95.6	NA		4	28	14.3	NA		0.5
Transgender man	1	136	0.7	NA		0	28	0	NA		0.6
Transgender woman	6	136	4.4	NA		1	28	3.6	NA		0.7
